# Long-term bevacizumab is safe and effective in managing small bowel angioectasias bleeding refractory to conventional treatments: a case report

**DOI:** 10.1093/gastro/goae070

**Published:** 2024-07-06

**Authors:** Debora Compare, Costantino Sgamato, Alba Rocco, Stefano Minieri, Sofia Cinque, Flaviana Giordano, Gerardo Nardone

**Affiliations:** Department of Clinical Medicine and Surgery, Gastroenterology, University Federico II, Naples, Italy; Department of Clinical Medicine and Surgery, Gastroenterology, University Federico II, Naples, Italy; Department of Clinical Medicine and Surgery, Gastroenterology, University Federico II, Naples, Italy; Department of Clinical Medicine and Surgery, Gastroenterology, University Federico II, Naples, Italy; Department of Clinical Medicine and Surgery, Gastroenterology, University Federico II, Naples, Italy; Department of Clinical Medicine and Surgery, Gastroenterology, University Federico II, Naples, Italy; Department of Clinical Medicine and Surgery, Gastroenterology, University Federico II, Naples, Italy

## Introduction

Small bowel angioectasias (SBAs) are vascular malformations composed of dilated and tortuous arterial or venous capillaries, accounting for 10% of all gastro-intestinal bleeding and 50% of small bowel bleeding, especially in elderly patients with co-morbidities [1]. A progressively aging population and the increasing use of antiplatelets explain the worldwide increase in SBA bleeding [2].

Treatment for SBA bleeding is challenging, as the small bowel is in the middle part of the digestive tract, so it can be difficult to explore. Different approaches such as endoscopic hemostasis, angiography with embolization, pharmacologic therapy (somatostatin analogues, oestrogen–progestagen, thalidomide), and surgery are used variably but there is no defined treatment strategy for refractory SBA bleeding [3].

Bevacizumab—a vascular endothelial growth factor inhibitor—has been shown to be effective in patients with hereditary hemorrhagic telangiectasia (HHT) and bleeding due to gastric antral vascular ectasia (GAVE) or SBA, recurring after endoscopic therapy. However, treatment dose and duration are not standardized and long-term effects are largely unknown [4].

We herein present a case of gastro-intestinal bleeding secondary to SBAs, refractory to endoscopic and pharmacological treatment with octreotide, treated with bevacizumab.

## Case presentation

A 69-year-old man was referred to our institution for severe anaemia with haemoglobin (Hb) levels of ≤67 g/L due to a 6-year history of recurrent SBA bleeding requiring multiple red blood cell (RBC) transfusions (at least twice/month) and intravenous iron replacement cycles. Medical history included type II diabetes mellitus, hypercholesterolemia, and arterial hypertension. Current therapy included atorvastatin, pantoprazole, metformin, and long-acting insulin. We excluded valvular heart disease and Osler–Weber–Rendu syndrome by using echocardiography and molecular testing (ENG gene), respectively. At presentation, the patient’s vital signs were normal. Physical examination was unremarkable except for mild pallor and moderate dyspnea on exertion. He had previously undergone SBA endoscopic treatments with argon plasma coagulation (APC) by double-balloon enteroscopy and had been on intramuscular long-acting octreotide 20 mg/month for 6 months. Capsule endoscopy showed multiple and large SBAs along the small bowel.

**Figure 1.**
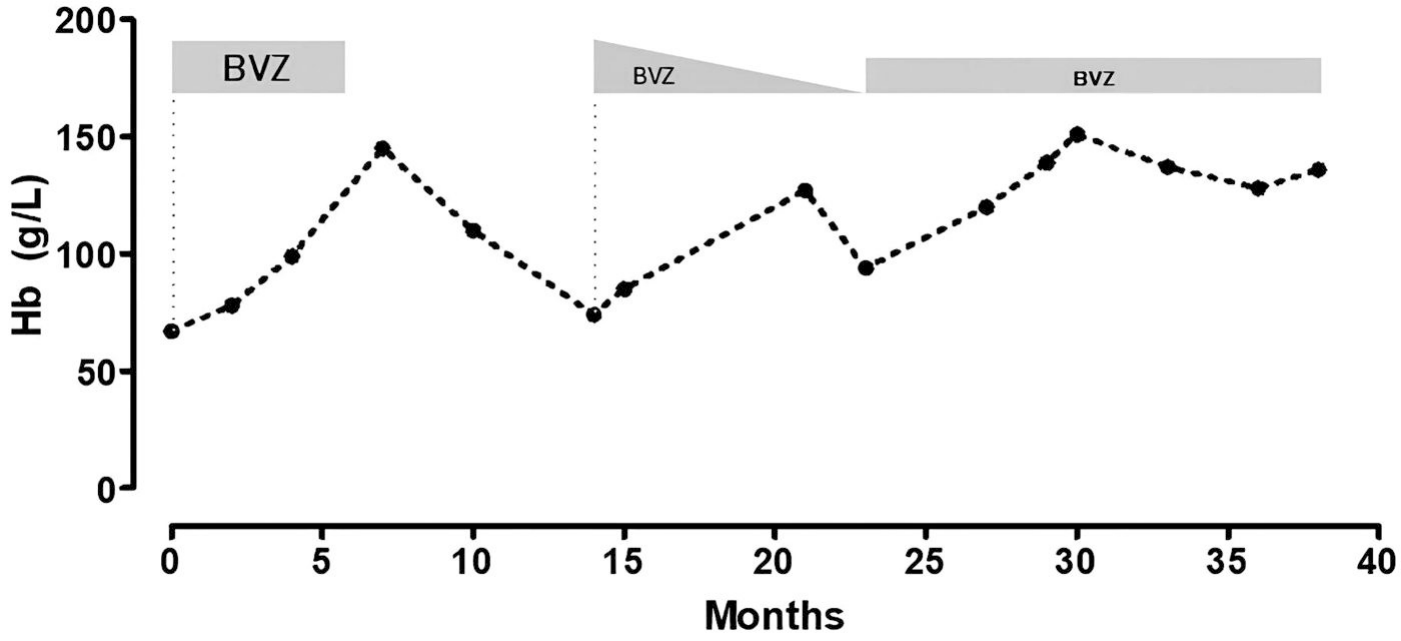
Mean Hb levels during the observation period. The grey thinner box corresponds to the lower dose of bevacizumab. Hb = haemoglobin, BVZ = bevacizumab.

We started intravenous bevacizumab at 5 mg/kg/2 weeks for 2 months followed by 5 mg/kg/month for 4 months. At the end of the treatment, there was a significant increase in Hb, with median levels rising to 113.5 g/L. However, 6 months after stopping therapy, Hb and ferritin levels dropped to 74 g/L and 13 ng/mL, respectively, requiring RBC transfusions and intravenous iron replacement. Thus, we started a new cycle of bevacizumab at 5 mg/kg/2 weeks for 2 months, followed by 5 mg/kg/month. After 4 months, we tried to use bevacizumab at 2 mg/kg/month but, within 3 months, Hb levels had progressively decreased to 94 g/L. Thus, we scheduled a maintenance treatment at 2.5 mg/kg/month. Capsule endoscopy performed at the end of treatment showed a significant reduction in the SBA number and size compared with baseline. Given the stability of Hb levels and no need for RBC transfusions or iron supplementation, we planned a maintenance protocol at 2.5 mg/kg/month, still ongoing for 3 years. Figure 1 shows the time-trend of Hb levels according to the bevacizumab schedule during the whole follow-up period. No adverse events were observed except for transient mild proteinuria of 232 mg/24 h and poor control of arterial hypertension requiring a therapy modification.

## Discussion

The estimated overall mortality from gastro-intestinal angiodysplasia bleeding is 2% per hospital admission, with lesions located in the small bowel having a worse prognosis [5]. The management of SBA bleeding depends on different factors such as the patient’s age and co-morbidities, as well as the type and frequency of bleeding episodes. The most effective treatment for SBA bleeding involves direct coagulation with APC via double-balloon enteroscopy. However, the rebleeding rate varies from 11% to 57% at 1-year follow-up due to untreated lesions not being detected or sporadically growing in other sites of the small bowel [1]. Medical intervention may play a role in the treatment of these patients [3]. Somatostatin analogues were associated with improved Hb levels, reduced transfusion requirement, and decreased rebleeding rates. However, more than one-quarter of patients were poor responders to treatment [6].

Currently, there is no established rescue treatment for refractory SBA bleeding and 10% of patients still depend on RBC transfusions and intravenous iron replacement, placing a significant burden on hospital resources [7].

Several case series reported a clinical benefit of bevacizumab in managing patients with HTT, GAVE, or SBA. Albitar *et al.* successfully treated 21 patients with intravenous bevacizumab for refractory GAVE/SBA bleeding and reported a significant reduction in the need for endoscopic procedures or RBC transfusions and an improvement in median Hb levels at 6–12 months of follow-up [4].

In our patient, bevacizumab was effective at controlling SBA bleeding refractory to endoscopic and medical treatment with octreotide, improving mean Hb levels and avoiding the need for transfusions. In HTT patients, most authors used bevacizumab at 5 or 7.5 mg/kg/2–3 weeks, but a lower dose of 1–2 mg/kg/3 weeks, which has the potential to reduce costs and adverse events, has also been employed successfully [8].

Another matter of concern pertains to adverse events, primarily the development of de-novo or worsening arterial hypertension and renal microangiopathic haemolytic anaemia with proteinuria. Other side effects included fatigue (10%), myalgia and/or arthralgia (6%), headache (4%), and venous thromboembolism (2%) [9]. Moreover, in patients with cancers, bevacizumab significantly increased the risk of all-grade and high-grade bleeding [10]. Our patient did not experience serious adverse events, but only mild transient proteinuria and worsening arterial hypertension. However, the causal relationship between these side effects and bevacizumab use cannot be definitely proved. Finally, despite its high cost, bevacizumab may reduce overall healthcare resource utilization and improve patient quality of life compared with standard-of-care treatment.

Notwithstanding these promising results, bevacizumab is far from being included in the therapeutic armamentarium for SBA bleeding, and the treatment dose and duration remain non-standardized. To our knowledge, this is the longest duration of bevacizumab treatment reported in the scientific literature for SBA bleeding.

In conclusion, bevacizumab may be a safe and effective treatment option for the long-term management of SBA bleeding refractory to previous endoscopic and pharmacological treatments. Thus, at the lowest possible dose, it could be considered an alternative rescue therapy in these challenging patients, ensuring close monitoring of side effects.
